# Contemplation by Design: Leveraging the “Power of the Pause” on a Large University Campus Through Built and Social Environments

**DOI:** 10.3389/fpubh.2020.00031

**Published:** 2020-02-28

**Authors:** Benjamin W. Chrisinger, Tia Rich

**Affiliations:** ^1^Department of Social Policy and Intervention, University of Oxford, Oxford, United Kingdom; ^2^Stanford Prevention Research Center, School of Medicine, Stanford University, Stanford, CA, United States; ^3^School of Medicine, Stanford University, Stanford, CA, United States

**Keywords:** contemplative practices, mindfulness, health promotion, workplace, higher education, colleges and universities, campus planning and design, case study

## Abstract

College and university campuses have long been designed as embodied places of societal values and aspirations, reflecting both academic traditions and heritages alongside social and scientific change and innovation. More pragmatically, these spaces share some commonalities with other living and working environments, and must adapt to changing technological and social norms. Since the 1970's, workplace adaptations included employer-sponsored health promotion programs and facilities. While campus environments such as fitness centers and dining halls have been incorporated into health promotion initiatives, other aspects of human well-being have been neglected. In this paper, we describe an initiative, Contemplation By Design, to incorporate contemplation and mindfulness into the daily lives of all members of the Stanford University community, including students, faculty, staff, and their families, as well as alumni and retirees who live close by. This case study highlights ways that physical planning and programmatic initiatives for contemplative practices have been integrated to deliver generalizable, community-based well-being resources that can be emulated in diverse settings throughout the Stanford University campuses, including the main campus and local satellite campuses. Based on experience drawn from Contemplation By Design, practical recommendations for designing contemplative practice spaces and programs are offered.

## Introduction

College and university campuses are amalgamations of built and natural environments where diverse groups of individuals live, work, learn, and play. For students, they can be both neighborhood and workplace. For employees, campuses are purpose-built workplaces that may closely resemble office, hospital, library, or dining spaces in non-academic settings. Indeed, in many communities, one or several colleges or universities can function as a primary employer, transport or housing provider, or landowner. Furthermore, many campuses engage in regular planning processes and develop land use documents akin to comprehensive plans. Scholars have also pointed to the role of American colleges and universities in the provisioning of public spaces (1–3), and as embodied places of societal values and aspirations, reflecting both academic traditions and heritages alongside social and scientific change and innovation (4). Thus, campus plans and designs often reach beyond the Ivory Tower and into surrounding neighborhoods and office parks, and, at least indirectly, follow an institution's alumni wherever they go after graduation. While the modern age of university planning is more characterized by the role of technology in the classroom, and broader questions about virtual online learning environments, the belief that good campus design can engender positive outcomes including inter- and intrapersonal personal values, emotional intelligence, and civic engagement, is resonant. Collectively, these outcomes can contribute to individual and community health and well-being.

### Background: Campus and Workplace Health Promotion Initiatives

The need to address rising rates of chronic disease through health promotion and prevention in community settings, rather than medical treatment of disease states in clinics and hospitals, has been noted by US researchers and policymakers since at least the 1980's (5, 6). As a type of community setting where young adults and adults spend the majority of their waking hours, schools and workplaces provide unique opportunities for health promotion, as administrators or employers may offer programmatic and economic incentives in addition to provisioning physical spaces and facilities to support individuals engaging in healthy behaviors (7, 8). Additionally, encouraging results from health promotion interventions embedded within worksites and schools, including higher education institutions, provide a further rationale for campus administrators to consider these initiatives for their own faculty, staff, or students (9–11).

Still, there is room for improvement. The Centers for Disease Control and Prevention's planning document Healthy People 2010 set a target of three in four employers offering a worksite health promotion program, though a 2004 national survey of worksites found that this was only the case among 6.9% of respondents (12). The more recent Healthy People 2020 goal is for 40% of employees to have access to workplace-sponsored stress prevention or reduction programs, which would represent a six percent increase from the 2010 baseline measure (13). Overall, the trend toward workplace health promotion is positive, with some promising returns in terms of lowered medical costs and increased productivity (10), though employer adoption and rigorous evaluation have fallen short of CDC aspirations (8, 9, 14). The provisioning of physical spaces for students and employees to practice healthy behaviors has been suggested as a means of further enhancing positive outcomes, though further research is needed (11).

### Campus Environments for Contemplative Practices

This case study highlights another impactful, but under-studied, element of campus design: *contemplative space*, or places that enable and/or encourage individuals to engage in any kind of *contemplative practice*. Contemplative practice (CP) is a term that encompasses a variety of behaviors that aim to quiet the striving mind, connect the individual to something larger than their own life, and sustain an experience of being seen, safe, soothed, and secure. A growing body of literature documents the social and biological benefits of contemplative practices, including stress and pain management, weight control, sustained physical activity, and promoting emotional health and overall fulfillment, purpose, and meaning (15). Contemplative practices have shown promise in reducing negative emotional behavior and increasing prosocial behaviors, thus allowing practitioners to build better social connections and feel part of something larger, purposeful, and meaningful (16).

In the clinical setting, studies have also demonstrated the benefits of contemplative practices for both patients and providers. For instance, a German study found better healing outcomes among hospital patients when their provider engaged in contemplative practices, even if the patient was unaware (17). One possible pathway between practitioners and patients may involve an enhanced sense of presence during interpersonal interactions that arises from their enhanced self-care (18, 19). Arthur Kleinman, a psychiatrist and anthropologist, describes shared presence as “an interpersonal process that mobilizes vitality from both clinician and patient, and from family caregiver and recipient of care” (20). Even outside of healthcare, there appears to be increasing interest in contemplative practices in the workplace, most notably yoga and meditation (21).

Research also demonstrates how contemplative practices can support an individual's discovery and cultivation of purpose and meaning in life, which is related to a variety of positive physical and social outcomes (22). For example, studies show that a strong sense of purpose is associated with positive health biomarkers, such as high density lipoprotein cholesterol (23–25), and behaviors, including better management of chronic conditions such as substance addiction, depression, and diabetes (26–30). More distal effects of purpose include reduced risk of all-cause mortality and cardiovascular events (31), and lower health care costs via greater use of preventive health services and fewer nights of hospitalization (32). Especially relevant to campus design, one longitudinal cohort study of college seniors found that cultivation of purpose and skills for sustaining that cultivation throughout life was significantly associated with generativity, personal growth, and integrity later on in adulthood (33).

Some evidence exists to suggest that reliably engaging with contemplative practices helps individuals develop positive personal and interpersonal traits. For example, individuals who regularly engage in contemplative practices have been shown to score higher on scales of prosocial and interpersonal traits (34), and a more recent meditation intervention found that the cultivation of purpose through meditation was essential to the success of the treatment group, relative to the control (35). While the literature investigating contemplative practices and the development of individual and community purpose or meaning is relatively nascent (36), academic leaders have previously underscored the creative social change-making potential of contemplative practices in higher education (37).

Just as workplace and campus health promotion campaigns are evolving to accommodate contemporary issues and interests, so too must modern campuses respond and adapt to the needs and values of their students. Recent studies have suggested that millennials may be expecting to see contemplative spaces, like meditation rooms, on campus (38). Campus planners and designers may also be confronted with needs to reimagine formerly on-campus religious buildings from single-denomination places of worship to multi-faith and/or multi-purpose environments or dedicating new structures or buildings to support new kinds of secular or spiritual contemplative and/or inter-faith activities (39). Whether by building anew or by repurposing existing environments, these on campus environmental supports may help administrators respond to calls to use contemplative practices as a means of achieving university goals, including teaching and learning outcomes (40, 41) and student health and well-being, especially mental health, and emotional well-being (42, 43).

## Context

Here, we offer a descriptive community case study of the multi-level, multi-year Contemplation By Design initiative at Stanford University as a model for enabling and encouraging campus staff, students, faculty, and their families to reap the benefits of contemplative practices. The case was selected based on our personal knowledge of the Contemplation By Design initiative, and we draw upon our experiences organizing and assessing its constituent programmatic elements to identify the key components (both in terms of built spaces and programs). The case study also incorporates program participation data from 2011-present to chart broader trends in interest and engagement in contemplative practices from community members, as well as historical antecedents related to the University's founding which help place modern conversations about supporting on-campus contemplative practices into a broader context. We illustrate how the initiative has encouraged campus stakeholders to leverage well-being opportunities afforded by the campus built environment (both existing and purpose-built) beyond those often implicated in traditional health promotion programs, which have tended to focus on athletic and dining-related campus environments. By describing the various program components and their interactions, we identify opportunities for other campuses to glean generalizable lessons for designing and implementing similar contemplative practice initiatives for community health promotion.

### Context for Contemplative Practices at Stanford University: Past and Present

Stanford University was founded as a deliberately innovative and unconventional, secular academic institution at the turn of the twentieth Century. Despite its modern notoriety as a center of high-tech productivity and innovation, the University's founders, the philanthropists Leland and Jane Stanford, insisted that the institution also seek to cultivate a sense of spiritual and religious purpose or meaning among the student population, breaking from many of the conventional norms among higher education that emphasized science and preparation for membership in the mechanistic endeavors of the Industrial Revolution. Following her husband's death, Jane Stanford, herself a multi-denominational Christian, continued to encourage the University's physical, and programmatic efforts at providing opportunities for contemplative, spiritual and religious exploration and discovery. Amid campus designing and planning, Stanford was particularly involved in shepherding the construction of a contemplative sanctuary as the key focal point at the center of Frederick Law Olmsted's campus plan for the university (44). Notably, this positioning of the sanctuary, known as Memorial Church, was criticized as it was non-sectarian and neither connected to a particular religious denomination, nor was it entirely secular and academic (44). Nevertheless, Jane Stanford maintained that “education needs intelligent guidance if it is to serve any good purpose,” and underscored this belief in an 1899 letter to the University's first president, “I have resolved to keep the University on the highest level morally and spiritually. The latter is more deeply interesting to me from the fact that it seems to be lost sight of in a sense” (45).

### Purpose-Built Contemplative Space at Stanford University

Modern changes or expansions to the campus built environment having included the deliberate provisioning of new spaces for contemplative practices, beyond the existing natural and non-denominational places of contemplation so integral to the original university plan. Recent campus projects reflect both the challenges and solutions developed by architects and planners to scale the principles of contemplative design to different site contexts.

#### Center for Inter-Religious Community, Learning, and Experiences (CIRCLE)

Opening in 2007, Stanford University's Center for Inter-Religious Community, Learning and Experiences (CIRCLE) was the first in a series of modern contemplative place-making efforts, and includes meeting spaces, an interfaith sanctuary, as well as office space for related student groups and administrators (46). Designed to be inclusive to all faith traditions and practices, CIRCLE is described as a “a welcoming space where religious and spiritual communities can deepen understanding of one another and find common ground together while embracing the particular aspects of their traditions and practices” (47).

#### University Contemplative Center

After an extensive planning and design process, Stanford University opened the Windhover Contemplative Center to the campus community in 2014, responding, in part, to calls to substantively address increasing student stress in the fast-paced context of the Digital Age (48). The Center and its grounds were deliberately planned to provide a secular, minimalist, and contemplative practice-designated space for the Stanford University community, as a compliment to existing religious and spiritual places on campus, like Memorial Church. Embedded within a grove of live oaks, the Center was carefully designed to incorporate aspects of light, shade, nature, and quiet, and is maintained as a “no-noise, no-work,” “technology-free,” space, without any available wireless internet connections. The Center also features a granite masonry labyrinth for the practice known as labyrinth-walking which facilitates spiritual centering, contemplation, and prayer, and hosts free guided meditation and mindful yoga classes that regularly fill to capacity.

#### Lucile Packard Children's Hospital

Recent rebuilding and modernization projects at the Children's Hospital of Stanford University have included distinct elements of “healing” design. Five gardens and 3.5 acres of green space are accessible to hospital patients, visitors, and staff, pursuant to clinical research showing that access to green space can be beneficial to patient outcomes (49), which Stanford University was early to adopt in the hospital's first iteration during the 1990's (50). Beyond its natural elements, contemplative spaces also feature prominently into the hospital's design. An outdoor labyrinth provides patients and visitors an opportunity to practice contemplation, as does a first floor sanctuary space. For healthcare providers, a staff-only garden is available as “a dedicated zone for mindfulness and stress reduction” (51).

#### Redwood City Campus Contemplative Space

A major campus expansion in nearby Redwood City, includes a designated contemplative space, that acknowledges the need for spaces where employees can engage in a variety of contemplative practices, particularly at a location more geographically constrained than the planned open space environment available on the main campus. The Redwood City Campus contemplative space offers both formal and informal practice periods in which instruction in meditation is offered, a group practice is held weekly and opportunities for contemplative movement and prayer or personal meditation practice are available seven days a week throughout the day. Meditation cushions are provided in a serene atmosphere with a glass wall that opens to a quiet garden, in which a portable roll-out labyrinth is available for use. Occupant feedback from Redwood City Campus staff, including perspectives on the functioning and use of the contemplative space, is being actively solicited by the university architect's office and Contemplation By Design staff in order to respond to and accommodate its effective use into the future.

## Contemplation by Design: Key Programmatic Elements

In 2013, Contemplation By Design was created and launched in preparation for and coordination with the 2014 opening of the Windhover Contemplative Center (48). The initiative augments longstanding health promotion programs for faculty, staff and for students, and is a campus-wide, multidisciplinary resilience-building health and well-being program that promotes the practice of contemplative lifestyle behaviors and incorporates the AMSO (awareness, motivation, skills and opportunity) framework for effective community health promotion (52–55). Faculty, staff, students and members of the greater University community are united through the opportunities to pause from high levels of productivity and innovation to experience multi-faceted, transformational learning, and develop skills to support sustainable, whole-hearted, ethical, purposeful engagement in all areas of research, teaching, learning, and service (56).

**Figure 1:**
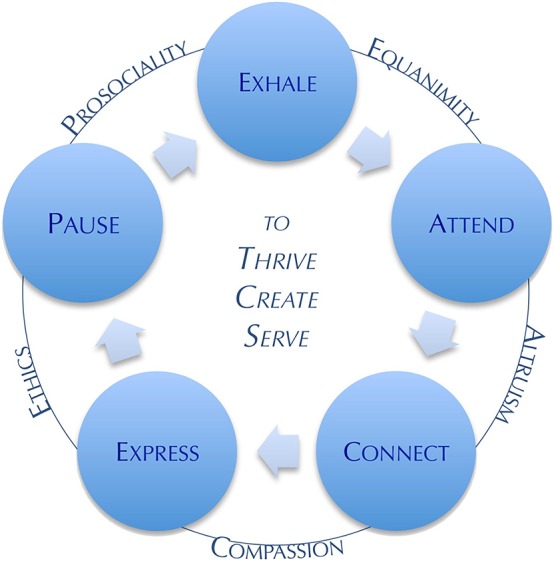
Contemplation By Design PEACE™ Paradigm. Ten interconnected constructs of contemplative practice to cultivate both state and traits of contemplation.

Contemplation By Design is guided by a paradigm of 10 interconnected contemplative practice constructs, the “PEACE™” paradigm (see Figure 1). Participants gain contemplative knowledge, first-hand experience, and skills to: pause the striving, driving, analytical, dualistic, and conceptualizing mind; cultivate spaciousness and possibility; sustain equanimity; and enter into compassionate relationship with themselves, others and nature; and be in a unified, interconnected oneness. They develop skills for sustaining the “power of the pause,” including deep breathing, intentional rest of the sympathetic nervous system and activation of the parasympathetic nervous system, revitalization of mind-body-spirt, and alignment between values and lifestyle habits. These skills are rooted in emerging evidence on the diverse benefits of contemplative practices, including self-awareness (57), attention regulation (58), neuroplasticity (59, 60), resilience, stress management, meaning and purpose, happiness, and compassion (58, 61–67).

Examples of Contemplation By Design programing include: lectures on contemplative neuroscience; skill-building classes; customized departmental workshops and professional trainings on contemplative practices, resilience, meaning and purpose; an annual 10 days free Summit; contemplative concerts; a website “hub” for contemplation resources such as: research articles, free online audio and video instruction, and a calendar of campus contemplative practice groups/opportunities; as well as consistent messaging from campus leaders to increase community-wide understanding of how to incorporate contemplative practices behaviors into daily life (see Table 1). Assessment is offered to participants through online self-report surveys on health and lifestyle habits, health status and circumstances, and includes questions about contemplative practices, stress level, ability to manage stress, positive states, depression symptoms, and sleep.

**Table 1:** Summary of Contemplation By Design components according to AMSO framework.

**Element**	**AMSO domain**			
	**Assessment**	**Motivation**	**Skill-building**	**Opportunities**
**Faculty/staff employee benefits**				
Quarterly contemplative practice classes			X	X
Free drop-in contemplative practice classes at main campus Contemplative Center and satellite campus Contemplation Space			X	X
Training/development funds-eligible contemplative practice classes			X	X
Customized departmental workshops			X	X
Employee incentive-eligible behavior change contemplative practice classes		X	X	X
**Academic offerings for undergraduate and graduate students**				
Contemplative practice-related courses for undergraduate/graduate students			X	X
**Free offerings for entire community of faculty, staff and students**				
Contemplation By Design Annual Summit				
P.E.A.C.E.™ Contemplative Concert				X
Community-wide “contemplative pause”		X	X	X
Lectures and presentations by campus leaders and experts		X		X
Skill-building workshops led by contemplative practice teachers from inside/outside University		X	X	X
Free drop-in contemplative practice classes at main campus Contemplative Center			X	X
Contemplation By Design participant feedback/evaluation survey	X			
Contemplative practices section of University's Well for Life Survey	X	X		
Contemplative practices section of University's Health and Lifestyle Assessment (a Health Risk Appraisal instrument)	X	X		
**Contemplation By Design website**				
Hub for information on free/fee-based contemplative practice-related trainings/courses and academic classes			X	X
Archived videos of Contemplation By Design programs		X	X	
Links to research on benefits of CPs		X		
Links to assessments of CPs	X			

### The Contemplation by Design Summit

The high-profile feature of Contemplation By Design is an annual, multi- day Summit, which provides an array of opportunities for students and community members to experience and broaden their understanding of contemplative practices. Summit programming offers a variety of secular contemplative practices, as well as cultural and spiritual examples. Workshops, seminars, lectures, a large concert, and a coordinated and campus-wide “contemplative pause” are all key components of this annual Summit (see Table 1). The number of Summit programming days and opportunities have increased year-to-year, from 23 events spread over 5 days in 2014, to 111 events over 10 days in 2019. Summit enrollments have increased nearly 5-fold over the same time period (*n* = 1,699 in 2014, *n* = 8,465 in 2019), and have coincided with an increase in enrollments in contemplative practice-related workplace wellness courses for faculty and staff and contemplative practice-related academic classes for undergraduate and graduate students (see Supplemental Figure 1). This trend could also indicate how individuals who are relatively new to contemplative practices during the Summit subsequently turn to workplace wellness or academic courses for deeper, multi-week engagement in the contemplative practice topic and skill-building.

Data from annual Summit online participant surveys (distributed via email) also provide insights into how Contemplation By Design programming has engaged new audiences. Of 2018 Summit respondents (*n* = 391), 29% reported that they had never previously done a contemplative practice before participating in the Summit, 62% reported that they learned at least one tangible skill that they intended to do in their daily life, and 70% reported an intention to incorporate information from the Summit into their daily life. Similar patterns were also found in Summit evaluations from earlier years.

Summit survey also provide guidance for improving programming design and content. For example, Summit participants are asked: “Why have you never tried taking a contemplative pause for relaxation and self-renewal before?” Top responses in both 2014 and 2019 included not knowing how to take a contemplative pause, not knowing about health and well-being benefits associated with contemplative practices, and having other means of relaxing or taking a quiet break (see Supplemental Table 1). These data support the value of providing contemplative practice skill-building opportunities to engage new people in these lifestyle behaviors and also providing ongoing opportunities and spaces for regular contemplative practice. Open-ended responses to the Summit evaluation survey along with open-ended class feedback and reflections provide additional context for how programs influenced participant perspectives on contemplative practices. Respondents mentioned the importance of the Summit in realizing opportunities to use the “power of the pause” on campus and elsewhere, and to connect with other members of the community (see Supplemental Table 2).

## Discussion

From our experience designing and implementing the Contemplation By Design initiative, several insights on creating a culture of contemplative practices on campus can be drawn for application in new settings. Here we offer some reflections on the connections between contemplative practice programming and place-making (Table 2), drawn from our experiences implementing contemplative practice programming in both purpose-built, retrofitted, and natural spaces. Based on these insights, we also suggest several specific design principles for new, retrofitted designated contemplative spaces, or repurposed spaces for temporary use for contemplative practice (Table 3), as well as reflections on the programmatic aspects of Contemplation By Design.

**Table 2:** Features of on-campus contemplative practice programming and place-making, based on the Stanford University experience.

**Contemplative programming**	**Contemplative place-making**
• Demonstrates the potential benefits of contemplative practices in everyday life, whether at work, in the classroom, at home, or anywhere in between. • Teaches accessible and scalable methods for doing contemplative practices • Builds a community buy-in and consensus that contemplative practices are valuable for individual and community well-being on campus and in the workplace • Drives demand for contemplative spaces in existing and future campus buildings • Creates ripple effects beyond the session: can encourage a connection between purposeful engagement to serve humanity and cultivating self-care • “Compassion received from people, practices or policies cultivates self-compassion and sustains compassion toward others which nourishes compassion in others” [(removed for peer review), 2017]	• Provides diverse points of entry for trying out contemplative practices (e.g., walking a labyrinth in a public space, attending a class in a designated meditation room or in a repurposed conference room or classroom, eating in silence at the designated tables during a mindful meal program) • Can be activated or re-activated by contemplative programming (e.g., guided tours of Memorial Church, campus natural spaces) • Builds upon existing campus assets, and reminds community members how they can continue to engage with what already exists (e.g., natural spaces or small garden areas for contemplative walks, meditation session at outdoor sculpture, reserving a lactation room for contemplative practice when it is not needed for its primary purpose) • Provides physical indication of institutional commitment to contemplative practices as meaningful health behaviors • Communicates compassion to users/inhabitants • Offers opportunities for contemplative practices to enrich dimensions of compassion, including purposeful engagement

**Table 3:** Practical principles of contemplative spaces, based on the Stanford University experience.

**Principles**	**Description**
Size	Nine square feet per person;^a^ Ceiling height of eight feet or more, optimally vaulted.
Nature	Views to green space, reflecting pool or quiet water element; Plants that reflect seasonal changes. Access to adjacent green space. Doors and windows can allow sounds (and even smells) to be integrated by incorporating courtyards or gardens, including moving water, and/or allow the flow of the wind.
Air and ventilation	Fresh air, operable windows, radiant heat instead of forced air system Cross ventilation for flow of fresh air through the room is fundamental since contemplative practices all use breath as the basis for their development. Ideally one opening receives the prevailing winds, and a second larger opening is on the opposite wall.
Illumination/lighting	Emphasize natural light, yet do not have it fall directly on people from skylights. In the case of yoga direct connection to the sun is integral to many exercises. Sheer window shades prevent glare. Warm indirect adjustable artificial lighting for use when needed.
Focus	Empty or minimal artwork (if any art, preferably color field, non-narrative images), no wireless internet access, to enhance opportunity to meet own mind, body, and spirit
Flooring and furniture	Wooden flooring to enhance connection to nature and to prevent dust accumulation in carpeting and rugs; available meditation cushions (zafus) and mats (zabutons), straight chairs, soft cushions for ergonomic support as needed on zafus or in straight chairs; open floor plan to provided ability for users to sit or lie down; no desks, which could be used for working
Signage	Written guidance for P.E.A.C.E.™ process and various contemplative practices that can be done in the space; Clear statements regarding the intention for the contemplative use of the space, especially in multi-purpose spaces (e.g., lactation rooms can be used for contemplative practices when not occupied for lactation). If space is not internet-free, then provide QR codes to the Contemplation By Design guided practices audio file resources
Mix of contemplative practice opportunities	Sensory garden, labyrinth, Japanese garden, meditation cushions, yoga mats
Programming	Peer leadership training and resource material for community member led practice groups; Equal allocation for resources to eating well, physical activity and contemplative practices

a *The meditation mat (zabuton) that a meditation cushion (zafu) sits on top of is ~30 “long by 28”. Adding space between individual cushions means adding 8–10 inches all around for a total space needed of 38–40" by 36–38; Roughly 9 square feet per person*.

### Integrating Contemplative Programming With Contemplative Spaces

Glimpses of the founders' success in cultivating a sense of meaning through campus design are evident around the present-day university's use of original built spaces such as Memorial Church, as well as its open spaces which are explored as potential sites for contemplative practices as part of Contemplation By Design programming. The initiative also encourages community members to envision the use of built spaces around the campus, including, but also beyond those for religious practices (e.g., Memorial Church or CIRCLE), or even those non-religious spaces specifically designed for contemplation (e.g., Windhover Contemplative Center). Adaptive options for contemplative practices are also encouraged throughout existing campus buildings by the designation of lactation rooms as “Lactation/Wellness Rooms” wherein nursing mothers have priority, though all community members are able to use the spaces for contemplation.

By encouraging participants to consider how to practice contemplation in environments that are conducive, but not always dedicated to contemplative experiences, such as prayer, yoga, or meditation, Contemplation By Design helps participants see themselves as competent contemplative practitioners in a wide variety of settings. In annual Summit feedback, participants have also described how the combination of built environment for contemplation and programming promoted their contemplative practice as a lifestyle habit in the same way physical activity programming and gyms promote a physically active lifestyle. Common space characteristics cited as supportive to contemplative practices included quiet and calmness, radiant heating and natural elements, with clear designations of space use for rest rather than for work, no technology, and integration into broader campus life (see Table 4). Some participants made comparisons to gym facilities, indicating that contemplative spaces were needed to support overall well-being, just as recreation centers are made available for physical activities.

**Table 4:** Contemplation By Design participant comments on effective qualities of contemplative practices spaces.

**Contemplative space principle**	**Summit participant feedback**
Quiet, no noise	“People usually stop speaking immediately after entering the room. This helps keep the stillness in the room.” “I work in the hospital where there is constant noise from the amazing, but noisy medical monitoring equipment—beeps, whirls, fans. In this (contemplation) space, I come into life giving silence so I can go back to save lives.” “…it's the one place that I'll find quiet on campus. No music playing, no phones ringing, no text messages beeping, no email alerts blinging, no leaf blowers or trucks backing up throbbing in my head, no key boards clicking. Just me and other people breathing.”
Calmer than rest of campus	“…it's the one place that I can go that I know that it's calmer than the other places, it's the one place that I'll find quiet on campus.” “In here I feel held, like I am in a green house built to shield me from harsh elements so I can grow until I am ready for raw exposure to full-blown weather. I can build my roots in here.” “Peoples stuff is not spewn about. In meetings people have their bags, clothes, papers, computers, water bottles, snacks all tumbled all over the place. In the contemplative center, there is a storage space near the entrance, so in the main it is quiet, free from all their stuff. It is refreshing. Not only are the people not speaking, their stuff is not shouting at me.”
Radiant heat	“My Tibetan heritage notes that drafts and wind, or Loong, can agitate the mind and add to anxiety and insomnia. Here (the designated contemplative center) the radiant heat in the floor creates a warm, quiet and calm that supports my meditation. I go to some of the other buildings with radiator heat to meditate too. It is calm there, and sooths me so I have less confused attachments and desires. With no wind blowing from air ducts, I am more clear headed and content.” “It is the quietest place on campus. There are not noises from air conditioning or heat vents. I can hear myself breathe.”
Intentional rest, No work	“Everywhere on campus people constantly work—on computers at meals, while at football games, even typing on laptops or talking on phones while in bathroom stalls.” “The contemplation building says ‘contemplation matters', it is OK to stop the constant human ‘doing' and to pause to begin the human ‘being'. Land is valuable and the fact that some was set aside for contemplative pursue says this institution values ‘being.”
No technology	“Having a place I can go; a dedicated no tech space is fantastic because every other place today is a technology zone. When I do contemplative practices like yoga or meditation I tune in to what's inside, instead of focusing what is going on around me, on the deliverables on my to do list. Technology pulls me out, to my to dos, to social media post about what other people are busy doing.” “Friends talk about wanting an embedded phone in their head—one of them may even invent it 1 day. But at the (contemplative center) there are no phones. You get to see the human head free of that appendage! I almost never see that anywhere. Somehow I actually feel more connected to them when there is no phone next to their head, even though we don't say a word to each other.”
Integrated with campus life	“In mediation, I watch, or try to, my busy mind, non-judgmentally, mindfully, compassionately. In here, sitting in a building that's at the heart of campus life, I can also watch the incessant business of this place, through the glass walls. The windows give me a lens on the life lived here. I can see myself in the people whizzing by on their phones, on bicycles, on foot, on paths and roads, even through the trees. When I go back out there I take a little of the calm from in here with me— and a little of ‘the watcher' with me.” “When I mediate in a classrooms set up for Guided Meditation, I feel like a scuba diver with a shielding lens that lets me look at the sea of agitated activity around us. The ‘pop-up zendo' creates a warm safe space where I can stand on dry land, a part from that sea. When I have class. Like now I have calculus in that same room, I remember how I felt when I was in the ‘pop-up zendo'.” “It (Memorial Church) is the biggest building on campus. It is one all the picture of campus. I had never cone in because I am not religious. Now that I did yoga in there during the Contemplation Summit I see it as a resource but I am not religious.”
Natural elements	“[The Contemplative Center] has a tree from the outdoor garden that sticks through the roof, bringing nature into the building itself.” “Impermanence is a theme in a lot of contemplation. The rammed earth walls of this building remind me that from earth I came and to earth I will return.” “I like that the colors of this place are earthen and flow into colors of what I look out outside the windows. The furniture and inside building design does not distract me to focus on them.” “I like the glass. Inside it is quiet while I watch the birds enjoy the fountain or reflecting pool. I can see the seasons change on the trees.” “Daytime classes do not have any artificial lights on. When we mediate the light in the room changes when the clouds pass in front of the sun. I like feeling like I'm outside, even when my eyes are closed.”
“Recreation Center or Gym” for the Whole Human Being—Mind-Body-Spirit	“(The designated contemplative center) is like a gym for contemplative practices. It offers a building; a place I know I can go for what I want to do.” “Like a gym, the contemplation center, is studio, like a dance studio or weight room. It offers a space for me to do contemplation on my own or with other people, in classes. It makes it easy to do regularly. I could do it on my own other spaces, but I'd make excuses like it is too noisy in my office, or I'll be interrupted and just go back to doing work. When I'm at the gym people know I'm there to exercise. When I am at the contemplation center people know I'm there to be fully human—to care for my whole self.” “I work out regularly at the gym across the street. I now go to the contemplation center after my work out. It helps me to be healthier— move my body, still my mind and peace fill my spirit.”

Health promotion and disease prevention programs in other arenas have demonstrated that individuals can develop enduring lifelong health habits through structured engagement with healthy behaviors through formal means for cultivating a habit, such as physical activity class and healthy meals programs in worksites (68–70). For instance, for an individual to increase their physical activity, they may be asked to engage in immersive physical activity experiences, such as fitness classes that use specialized instruction or equipment only available at a gym; yet, a participant may also be asked to consider complimentary behavior changes, such parking further from one's destination to incur a short period of walking. Similarly, a contemplative practitioner may feel the need to immerse themselves in a meditation class at the Contemplative Center, though they could also adopt a contemplative practice as part of their daily train or bus commute to campus. Further research exploring the transportability of contemplative practice skills beyond designated contemplative spaces is warranted.

### Design Principles for Contemplative Spaces

Design organizations have only begun to develop guidelines and certification systems for contemplative spaces. For example, the International WELL Building Institute has proposed a building standard that includes a “Mind” concept, which identifies factors such as altruism, healthy sleep policy, and biophilic design (71). Researchers have outlined general categories of “contemplative landscapes,” based on expert review and elicitation, which include landscape layers, landform, vegetation, light and color, compatibility, archetypal elements, and character of peace and silence (72). Still, these guidelines for mind-friendly workplaces do not completely address contemplative practices, nor do they describe the ideal form for specific, dedicated spaces for them to occur. With this in mind, further study of the influence of building and landscape architecture on contemplative practices is warranted.

Here, we reflect on effective ways of (re)dedicating (as in the case of Memorial Church) or creating contemplative spaces (as with the Contemplative Center), and identify 10 practical principles for contemplative spaces, drawn from our experiences designing, and implementing contemplative practice programming at Stanford University (see Table 3). We believe that principles of size, ceiling height, nature, ventilation, illumination, focus, flooring and furniture, signage, mix of contemplative practice opportunities, and programming have all influenced the relative success of different contemplative spaces around the university. These principles may be adaptable to a variety of contexts, from smaller colleges and universities, to large employers in office campuses.

### Contemplative Practice Programming

The AMSO model for community health promotion suggests an optimal programmatic framework that reflects the relative influence of constructs to achieving health behavior change: 5% awareness, 30% motivation, 25% skills, and 40% opportunity (52, 53). Survey data from Contemplation By Design show how in 2014, 50% of respondents reported that the primary benefit of the initiative was the provisioning of opportunities to engage in contemplative practice (see Supplemental Table 3). This figure declined over subsequent years (to 28% in 2018), perhaps suggesting that once opportunities were built, literally in this case in terms of providing several new designated spaces and a reliable schedule of space repurposed for contemplative practices, along with programming offering a variety of high-quality contemplative practices skill-building classes, perceived benefits of participation shifted to other domains. Further research and program evaluation is needed to illuminate the most effective mix of programming for promoting contemplative practice behaviors. By offering multiple doorways, for literal and figurative entry, into the benefits of contemplative practices health promotion programs can effectively provide opportunities that appeal to a wide range of people's existing interests and life goals.

To date, much of the contemplative programming has focused on neuroscience, emotional health and well-being, or spiritual seeking. Contemplative practices also are keenly relevant to facilitating integrative learning, sustainable careers in public service, acts of social justice and democratic values. By thinking broadly about contemplative practice programming and places to nourish contemplative skills, public health policy and initiatives can utilize variety of existing spaces to facilitate experience of contemplation as something that is adaptable and transportable, and can simultaneously promote the health of others, as well as oneself.

### Limitations

While we have attempted to identify relatively unique features of the case context that might limit the generalizability of themes identified to other settings, it is possible that other unidentified factors have influenced the success of Contemplation By Design initiatives. Further research of similar programs in other contexts could help test or validate the robustness of the suggestions we have made. Additionally, new analyses of individual-level participant data could start to unpack specific programs, activities, or spaces that were particularly effective in supporting learning and behavior, with special attention to possibly influential participant characteristics (e.g., age, employee/student status, prior experience with contemplative practices, etc.). By providing a descriptive overview of the quantitative and qualitative insights gleaned from program assessments, we hope to have provided a general framework for others to design similar or improved evaluation opportunities.

## Conclusion

In this period of internationally and historically high rates of chronic disease, especially stress-related disease, we also see increasing (or, perhaps, re-emerging) interest in contemplative practices such as meditation and yoga. The field of health promotion is ripe to integrate contemplative practice into community-based program design as society responds to mounting stressors and dissonance, and grows toward what nourishes it, including secular opportunities for contemplative practice and self-care (21). Campus designers and planners can respond to these forces by facilitating an ecology of designated or repurposed contemplative space, recognizing the value of these places to help individuals develop contemplative skills for effective stress management and enhanced capacity to constructively engage with the complexity of the modern life and world issues that increasingly impact all of us each day. Contemplative practices and spaces offer an important cost effective addition to public health policy, programing, and as a tangible resource essential to an environment designed to promote health and well-being.

As has been demonstrated in other lifestyle behaviors, significant advantages for human health can be made by leveraging what is known about effective health promotion and architectural and urban design. Adding contemplative practice behaviors to this mission has great potential to promote broad well-being, both at the individual and societal level, and research shows that contemplative practices both directly and indirectly contribute to enhanced health and well-being (73). Stanford University has leveraged both programmatic and environmental resources to provide its students, faculty, staff and community members with deep and broad opportunities to engage with contemplative practices. While some university initiatives, such as the construction of Windhover Contemplative Center, are not readily replicable elsewhere, we offer adaptable principles that may be applied in a variety of campus and “campus-like” environments. Overall, our experience suggests that contemplative places and programming can contribute to a community-wide cultural shift toward recognizing the “Power of the Pause.”

## Data Availability Statement

All datasets generated for this study are included in the article/Supplementary Material.

## Author Contributions

BC and TR contributed to the conceptualization of the paper. BC prepared the manuscript for publication. TR provided detailed comments and feedback.

### Conflict of Interest

The authors declare that the research was conducted in the absence of any commercial or financial relationships that could be construed as a potential conflict of interest.

## Supplementary Material

Figure S1 Increases in Contemplation-Related Programming Enrollment at Stanford, 2011-2018. Note: Contemplative Practice-Related Healthy Living Course enrollment is limited to Stanford faculty and staff; *Contemplation By Design* Summit enrollment is open to faculty, staff, students, and community members.
