# Supplementary figures and images for: Contemplation by Design: Leveraging the “Power of the Pause” on a Large University Campus Through Built and Social Environments
Source: Front Public Health. 2020 Feb 28;8:31. doi: 10.3389/fpubh.2020.00031 (PMC7059735; doi:10.3389/fpubh.2020.00031)

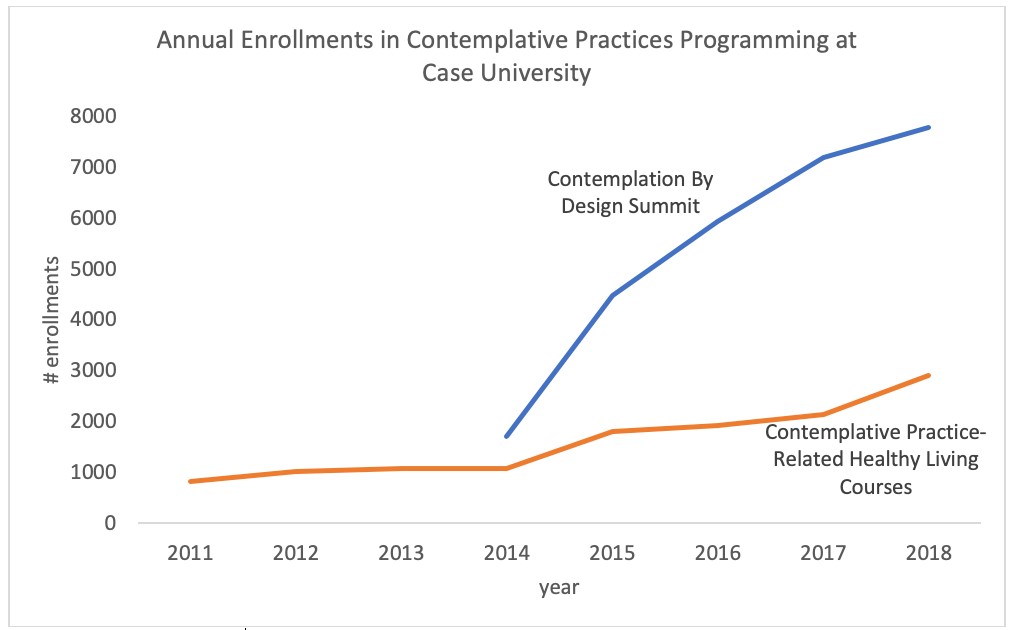

Supplement: Figure S1 — Increases in Contemplation-Related Programming Enrollment at Stanford, 2011-2018. Note: Contemplative Practice-Related Healthy Living Course enrollment is limited to Stanford faculty and staff; Contemplation By Design Summit enrollment is open to faculty, staff, students, and community members. [file Image_1.JPEG]
