# Supplementary material for: Contemplation by Design: Leveraging the “Power of the Pause” on a Large University Campus Through Built and Social Environments
Source: Front Public Health. 2020 Feb 28;8:31. doi: 10.3389/fpubh.2020.00031 (PMC7059735; doi:10.3389/fpubh.2020.00031)
Supplement: Supplementary file 2 [file Table_1.docx]

**Supplemental Table 1.** Post-Contemplation By Design Summit characteristics and participant feedback, 2014 and 2019

|  | | *Year* | |
| --- | --- | --- | --- |
| *Summit characteristics* | | **2014** | **2019** |
| Summit length (days) | | 5 | 10 |
| Total sessions offered | | 23 | 111 |
| Total enrollments* | | 1,699 | 8,465 |
| Total survey responses | | 221 | 391 |
| *Question: Why have you never tried taking a contemplative pause for relaxation and self-renewal before?* | |  |  |
| 1 | I did not know that taking a contemplative pause is good for my health and well-being. | 14% | 12% |
| 2 | I did not know how to take a contemplative pause. | 18% | 24% |
| 3 | I try to unwind by watching TV, checking Facebook, shopping, or talking on the phone instead of taking a contemplative pause. | 14% | 14% |
| 4 | Physical discomfort or pain. | 1% | 1% |
| 5 | Discomfort with being more aware of my thoughts. | 2% | 8% |
| 6 | Discomfort with being more aware of my emotions/feelings. | 3% | 8% |
| 7 | I take care of others more than I take care of myself. | 14% | 8% |
| 8 | Friends and family do not support me in taking time to take a contemplative pause or to meditate. | 4% | 2% |
| 9 | In the past, co-workers would not have supported me in taking time to pause or meditate. | 7% | 1% |
| 10 | I dance, play a musical instrument, create art, garden, or cook as my way of taking a quiet, contemplative pause instead of meditating or doing mindful yoga. | 19% | 14% |
| 11 | Other | 4% | 8% |
|  | Total | 100% | 100% |

*Note: Total enrollments reflects the number of registrations for various Summit programs; multiple enrollments per individual were possible in both 2014 and 2019.
