# Supplementary material for: Contemplation by Design: Leveraging the “Power of the Pause” on a Large University Campus Through Built and Social Environments
Source: Front Public Health. 2020 Feb 28;8:31. doi: 10.3389/fpubh.2020.00031 (PMC7059735; doi:10.3389/fpubh.2020.00031)
Supplement: Supplementary file 3 [file Table_2.docx]

**Supplemental Table 2.** Selected open-ended responses to the Contemplation By Design Summit evaluations based on themes of learning opportunities and connecting with community.

| **Learning Opportunities** | **Connecting with Community** |
| --- | --- |
| “Even as someone who is familiar with mindfulness, and meditates daily, I still learn many new things about contemplation!” | “I resonated deeply with the keynote. I shared some of the main ideas with my co-workers during our morning Huddle at work the following day.” |
| “I really appreciate this concentrated time for reflection and exploring contemplation. I like learning new techniques and the science that supports these ancient practices.” | “The flowers made available at the P.E.A.C.E. table was a nice touch to [*sic*]. I brought it home and gave me an opportunity to share with the family why I got an iris flower at work that day.” |
| “I simply was not aware of the various ways to take a contemplative pause. Now that I am, I’d like to try to incorporate it into my day in some way.” | "Also, I love seeing the community you know is there during this [Summit] week.” |
| “I found a lot of peace and enjoyment during my Contemplation By Design experiences [*sic*]. I think it was very beneficial because I am always stressing out about school and my duties playing on our varsity football team. I think pausing the rushing of my busy dynamic life to reflect on life really helped me. This is important for people of all ages, but especially important for people college age. College is just such a fast-paced lifestyle—from academics to athletics to social lives. It is good to just take a step back to reflect and enjoy how beautiful life is and how fortunate I am to be at a place as magnificent as Stanford. So I am grateful Stanford offers Contemplation By Design to help us slow down and be grateful— to learn contemplative practices and to enjoy our lives.” | “I love Contemplation By Design! It makes me very proud to work at Stanford.” |
| I’m much clearer about how various forms of meditation work and how they impact my mind and neurophysiology in general and can vary during each practice and over time. During the Contemplative Walk around the campus I saw I can take the practices anywhere with me, off the meditation cushion. The Summit talk on mind training really resonated, bringing into focus the big picture of what I was doing in the sequence of practices in the weekly guided meditation sessions: open awareness body scan, focused attention breathing, and the kind intention of self-compassion and compassion. | “The Summit’s talk with the monk and politician was a fascinating discussion highlighting the role of meditation in solving the world’s problems (climate change, racial injustice, depression, opioid addiction) by bringing people together to help each other with wise compassion. Another Summit speaker was funny, thoughtful, personal, pragmatic. His stubborn skeptical view of contemplative practice and meditation was so relatable and impactful. Ultimately, he tied together all that I have been thinking and thus reinforced why I want to commit to a contemplative practice — the enhancement of discernment, perspective, fulfillment, and kindness, both internally toward myself and externally toward others. All are so worth the effort of time spent doing meditative practice.” |
