# Supplementary material for: Contemplation by Design: Leveraging the “Power of the Pause” on a Large University Campus Through Built and Social Environments
Source: Front Public Health. 2020 Feb 28;8:31. doi: 10.3389/fpubh.2020.00031 (PMC7059735; doi:10.3389/fpubh.2020.00031)
Supplement: Supplementary file 4 [file Table_3.docx]

**Supplemental Table 3.** Post-Contemplation By Design Summit participant feedback: perceived support of programming for engaging in contemplative practices

|  | Year | | | |
| --- | --- | --- | --- | --- |
| *Which ONE of the following 6 types of programming would be MOST HELPFUL for supporting you to do this (contemplative practices) behavior more frequently* | 2014  (n=273) | 2016  (n=490) | 2017  (n=456) | 2018  (n=288) |
| Information on the FACTS about how this behavior is beneficial to my well-being. | 8% | 20% | 23% | 21% |
| Experiences that help me build my SKILLS for doing this behavior. | 20% | 26% | 26% | 28% |
| INSPIRATIONAL examples of how people have incorporated this behavior into their lives and how it helps them. | 10% | 20% | 20% | 23% |
| OPPORTUNITIES to do this behavior during a set time and location for doing this behavior either on my own or with others in a group. | 49% | 28% | 28% | 26% |
| ASSESSMENT^*^ of my behaviors and health status specific to this behavior. | 9% | N/A | N/A | N/A |
| I do not know | 4% | 6% | 3% | 2% |
| Total | 100% | 100% | 100% | 100% |

^*^ The ASSESSMENT item was removed from the survey after contemplative practice behavior items were incorporated into the Health Risk Appraisal regularly offered to the community.
